# Exploring the Pivotal Role of the CK2 Hinge Region Sub-Pocket in Binding with Tricyclic Quinolone Analogues by Computational Analysis

**DOI:** 10.3390/molecules22050840

**Published:** 2017-05-19

**Authors:** Yue Zhou, Na Zhang, Shan Tang, Xiaoqian Qi, Lijiao Zhao, Rugang Zhong, Yongzhen Peng

**Affiliations:** 1 National Engineering Laboratory for Advanced Municipal Wastewater Treatment and Reuse Technology, Engineering Research Center of Beijing, Beijing University of Technology, Beijing 100124, China; zhouyue2016@bjut.edu.cn; 2 Beijing Key Laboratory of Environmental & Viral Oncology, College of Life Science and Bioengineering, Beijing University of Technology, Beijing 100124, China; tangshan@emails.bjut.edu.cn (S.T.); 1992346688@emails.bjut.edu.cn (X.Q.); zhaolijiao@bjut.edu.cn (L.Z.); lifesci@bjut.edu.cn (R.Z.)

**Keywords:** protein kinase CK2, inhibitor, fragment-based design, tricyclic quinoline compounds, pharmacophore group

## Abstract

Protein kinase CK2 has been considered as an attractive therapeutic target of cancer therapy. The tricyclic quinoline compound CX-4945 is the first representative of CK2 inhibitors used in human clinical trials. The binding of non-2,6-naphtyridine substituted compounds **27e** (IC_50_ > 500 nM) and **27h** (IC_50_ > 1000 nM) to CK2 is abolished. However, the unbinding mechanisms due to the key pharmacophore group replacement of compounds **27e** and **27h** are unveiled. In the present work, combined computational analysis was performed to investigate the underlying structural basis of the low-affinity of two systems. As indicated in the results, the loss of hydrogen bonds between the non-2,6-naphtyridine and the hinge region destroyed the proper recognition of the two complexes. Besides, the allosteric mechanisms between the deviated ligands and the changed regions (G-loop, C-loop and β4/β5 loop) are proposed. Furthermore, energetic analysis was evaluated by detailed energy calculation and residue-based energy decomposition. More importantly, the summary of known polar pharmacophore groups elucidates the pivotal roles of hinge region sub-pocket in the binding of CK2 inhibitors. These results provide rational clues to the fragment-based design of more potent CK2 inhibitors.

## 1. Introduction

Protein kinase CK2, also known as casein kinase II, is a ubiquitous eukaryotic serine/threonine protein kinase [1,2]. The stable heterotetramers assembled by catalytic (α) and regulatory subunits (β) catalyze the phosphorylation of over 300 known substrates involved in important cellular processes [3,4]. Most substrates are transcription factors or regulatory proteins, by which CK2 is implicated in signal transduction pathways associated with human diseases [5,6]. The over expression of CK2α is particularly elevated in various malignant tumors compared with normal tissues or cells [7,8]. Consequently, CK2α is considered as a potential therapeutic antitumor target, and the discovery of ATP-competitive inhibitors has been the focus of anti-cancer drug design.

In recent years, numerous efforts have been made to design and synthesize a series of ATP-competitive inhibitors, including polyhalogenated benzimidazole derivatives [9,10], anthraquinone, tricyclic quinolone derivatives, natural products and others [11,12,13]. However, most inhibitors are precluded to be the drug candidates because of cytotoxicity, genotoxicity and other pharmaceutics deficiencies [14,15,16]. Surprisingly, with the favorable safety and pharmacokinetic property, the tricyclic quinolone derivatives CX-4945 has entered into human clinical trials and is also used in the treatment of cholangiocarcinoma approved by FDA [17,18]. Meanwhile, CX-4945 and CX-5011 have been demonstrated to overcome drug resistance in cancer therapy [19,20]. Therefore, tricyclic quinolone inhibitors are expected to be the anticancer drug candidates.

The typical binding pocket of CK2α is composed of hydrophobic regions, a positive area and a hinge region [12,21,22]. CX-4945, a representative inhibitor with holistic recognition mechanism, not only establishes interactions with hinge and positive regions simultaneously, but also shows higher inhibitory activity (IC_50_ = 0.3 nM) [23,24]. The tricyclic skeleton of the compound makes strong contacts with residues in the hydrophobic regions, which is a region of the protein that stabilizes binding to CK2. Meanwhile, the pyridine and carboxylate group of CX-4945 establish interactions with the hinge and positive regions, respectively. These polar interactions have a function in orienting the inhibitors in the binding pocket. In the previous studies, we have firstly built 3D-QSAR models for a series of tricyclic quinolone derivatives and identified two key pharmacophore groups: the 2,6-naphtyridine group and R2-carboxylate-substituent [25]. It was concluded that the simultaneous presence of these interactions is essential for guaranteeing the high potency of ATP-competitive inhibitors.

Experimental results also support the view that the binding modes of compounds and active site features are the most important consideration for successful CK2 inhibitor design. In our previous study, we have elucidated the structural basis for low-affinity binding of non-R2 carboxylate-substituted tricyclic quinoline analogs to CK2α using comparative MD simulations [26]. Compound **12** (IC_50_ = 0.5 nM) was chosen as a reference inhibitor owing to the polar interactions formed between R2 carboxylate-substitution and 2,6-naphtyridine group with the positive and hinge region, respectively. Results showed that both the changed conformation of CK2α and deviated orientation of ligands occurred in the two non-R2 carboxylate-substituted compound systems, which resulted in the inappropriate CK2α-ligands recognition and provided a structural basis for the decreased inhibitory activity. Notably, the alteration of 2,6-naphtyridine is also fatal to inhibitory activities of tricyclic quinolone derivatives. The comparison of the structure and inhibitory activity of compounds **27e** (1,6-naphtyridine) and **27h** (phenyl) versus compound **12** (2,6-naphtyridine)indicates that both the other substituent at the responding site and the position alteration of the key function result in a significant reduction of inhibitory activities (1000 and 2000-fold decrease) [27]. Therefore, there is an urgent need to systematically investigate the essential role of 2,6-naphtyridine on the inhibitory activities of tricyclic quinolone derivatives.

Nowadays, molecular dynamics (MD) simulations are useful and crucial tools in drug discovery [28,29]. MD has proved to be a useful complement to structural and other experimental studies in elucidating detailed dynamical behaviors processes at the molecular level [30,31,32]. In this study, computational investigations, including molecular docking and MD simulations were conducted to examine the detailed binding modes and remarkable conformational alterations of three systems. Also, molecular mechanics Poisson–Boltzmann and generalized Born/surface area (MM-PB/GBSA) binding free-energy calculations were conducted to elucidate the instability of the non-2,6-naphtyridine substituted compound systems. Our findings may provide valuable information for further structural modification and development of highly potent CK2α inhibitors.

## 2. Results and Discussion

### 2.1. Molecular Docking Analysis

**Figure 1:**
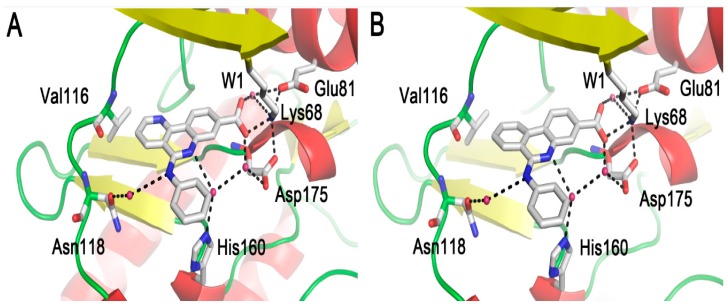
Binding modes of compounds (**A**) **27e** and (**B**) **27h** with CK2α based on molecular docking.

In previous studies, the successfully reproduced binding modes of CX-4945 and compound **12** to CK2α confirmed the validity and reliability of the docking method for tricyclic quinolone derivatives. As observed in compound CX-4945 and **12**, two non-2,6-naphthyridine compounds **27e** and **27h** located at the active site of CK2α primarily through hydrophobic and polar interactions (Figure 1). The tricyclic skeleton and phenyl moiety of compound embed in the hydrophobic regions I (Val53, Val66, Phe113, Met163 and Ile174) and II (Leu45, His115 and His160). Meanwhile, the carboxylate substituent, a negative charge center, created electrostatic interactions with the positive area involving the W1, Glu81 and Lys68. In addition, two water-bridged hydrogen-bond involving residues His160 and Asn118 also existed in two complexes.

Specifically, in contrast to compound **12**, the 1,6-naphthyridine of compound **27e** and isoquinoline of **27h** did not establish any polar interactions with Val116 as compound **12** did, and thus may have a negative contribution to the inhibitory activity of **27e** and **27h**. We speculate the loss of the H-bond of the 2,6-naphthyridine group with the hinge region exerts a fatal impact on the inhibitory activity of CK2 inhibitors. The analysis is consistent with relevant references [27] and may provide rational clues to the design of more potent CK2 inhibitors by considering the key functional groups and residues.

### 2.2. Molecular Dynamics Simulationstudies

#### 2.2.1. Overall Features of Dynamic Behaviors

**Figure 2:**
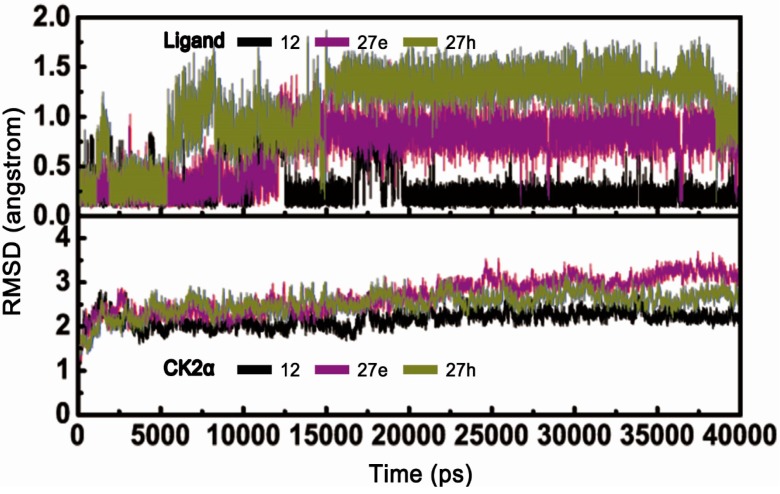
The time dependence of RMSD of inhibitors (upper) and CK2α (lower) for CK2 in complex with compounds **27e** and **27h**.

MD simulations of 40 ns duration were performed to explore the dynamic behaviors of the CK2α-**12**, **27e** and **27h** systems, respectively. For each trajectory, the time-dependent RMSD profile from the starting structure is shown in Figure 2. The fluctuations of the three systems followed the same trend in all cases throughout the simulations, and acceptable equilibrium plateaus were approached during the last 20 ns. As depicted in the lower panel, some instability could be observed for CK2α of other two systems (2.5 Å) in contrast to the CK2α-12 complex (2.0 Å). This indicates that the two instable systems underwent conformational changes to some degree. A fact worth highlighting is that three compounds exhibited significantly different behaviors observed in upper panel. The RMSD value of compound **12** remained around 0.25 Å during the MD simulation. In contrast, the RMSD values of compounds **27e** and **27h** gradually increased from 0.2 Å to 1.5 Å with the averaged values of 1.0 Å and 1.5 Å, respectively. It was speculated that two compounds may deviate away from their original positions and thus induce the inappropriate interactions of two systems.

**Figure 3:**
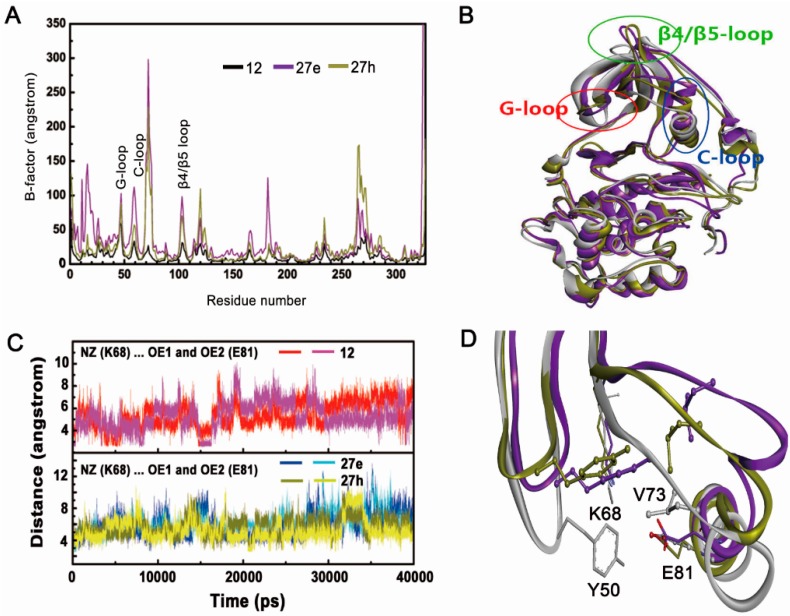
(**A**) Calculated per-residue B-factor of three complexes systems; (**B**) Superimposed average structures of CK2α–**12** (gray), CK2α–**27e** (purple) and CK2α–**27h** (brown) complexes; (**C**) Time evolution of distances between NZ atoms of Lys68 and OE1 and OE2 atoms of Glu81; (**D**) Coupled interactions between C-loop and G-loop in CK2α–**12** (gray), CK2α–**27e** (purple) and CK2α–**27h** (brown).

The residue fluctuations characterized by B-factors were evaluated to detect the flexible regions of the CK2 structure. It can be clearly seen that the C loop, G loop and β4/β5 loop had higher B-factor values (Figure 3A), suggesting that these residues appeared to exhibit abnormal behaviors in the enzymatic function of CK2α. Based on the structural superimposition of the three systems, some remarkable differences in secondary structure in contrast to the original configuration were clearly found (Figure 3B). These changes were distributed primarily over residues located in the β4/β5 loop, C-loop and G-loop region in the two complexes. This was especially in the “stretch” conformation of β4/β5 and open G-loop regions, which resulted from the disruption of allosteric mechanisms involving two pairs of hydrophobic interactions between Phe54 and Tyr50 of G-loop and Ile69 and Val73 of the C-loop, as well as the electrostatic interaction networks of Lys68 and Glu81 and W1 (Figure 3C,D). It was readily apparent that non-R2 carboxylate-substituted compounds follow the same allosteric mechanism as indicated in previous studies [26]. Meanwhile, similar coupling mechanism between the C-loop and the G-loop have also been used to interpret the loss of catalytic activity of cAMP-dependent protein kinase (PKA) [33] and cyclin-dependent kinase 2 (CDK2) [34], and glycogen synthase kinase-3 (GSK3) [35].

Besides the C-loop, G-loop and β4/β5 loop of two systems, other areas with higher B-factors also could be noticed in the Figure 3A, including residues located at N-terminal (Arg10-Val30) and C-terminal (Lys260-Arg280), residues from Glu55 to Val65 of linker-loop of C-loop and G-loop, and the hinge region (Ala110-Asp120). The N-terminal and C-terminal residues were exposed to the solvent and lacked of the anchoring interactions with neighbor residues, which resulted in a certain degree of flexibility. The reorientation of the G-loop and C-loop inevitably compelled the linker-loop residues (Glu55 to Val65) moving away from original position. In addition, the deviated compounds **27e** and **27h** induced conformational changes of hinge region. These speculations could be supported by the related references [25,26,31,36].

#### 2.2.2. Inappropriate Recognition Mechanisms

**Figure 4:**
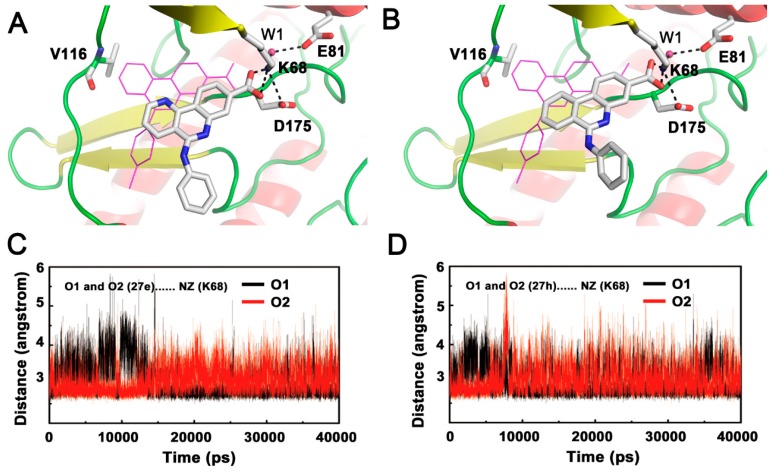
Stable binding mode of compounds (**A**) **27e** and (**B**) **27h** compared to compound **12** (magenta); Distances between NZ atoms of Lys68 and (**C**) O1 and O2 atoms of compound **27e**, (**D**) O1 and O2 atoms of compound **27h**.

The subtle difference between compounds **12**, **27e** and **27h** is only the alteration of the nitrogen atom in the 2,6-naphtyridine, which generates a significant decrease in inhibitory potency. Although the skeletons of compounds **27e** and **27h** located in the same active site observed for compound **12**, the orientation of two non-2,6-naphtyridine substituted compounds were shifted significantly and deviated from their original positions of docking poses. Specifically, the conserved H-bond with Val116 of the hinge region was severely destroyed; whereas the conserved carboxylate group still made stable polar interaction with the positive area residue Lys68, which could be confirmed by the distance between O1 and O2 atoms of compounds **27e** and **27h** and NZ atom of Lys68 (Figure 4).

Due to the incapability of the non-2,6-naphtyridine to form H-bond with hinge region, the skeleton of two inhibitors could not be fixed in the original position and thus migrated during dynamics simulation. Meanwhile, a strong electrostatic interaction network involving Lys68, Glu81, Asp175 and the carboxylate group make an extra contribution to the shift of tricyclic skeletons from the hinge region, which was followed by movement of the phenyl moiety toward the G-loop. We can speculate that the above may be the rational reason for the low inhibitory activities of non-2,6-naphtyridine substituted compounds. Thus, the simultaneous presence of polar interactions between inhibitors and the hinge region as well as the positive area is essential for recognition and location of this class of compounds. Interestingly, the same circumstances can also be occurred for the non-R2 carboxylate-substituted systems, which suggests that tricyclic quinoline analogs with the improper pharmacophore, non-R2 carboxylate and non-2,6-naphtyridine, follow the same inappropriate recognition mechanism for low-affinity binding of compound to CK2α [26].

Comparative analysis of known ATP-competitive inhibitors has confirmed that pharmacophore groups play a critical role to maintain the binding of inhibitors. For example, compounds CX-4945, pyrazolo[1,5-*a*][1,3,5]triazine derivative **9e**, and pyrazolo[1,5-*a*]pyrimidine derivative AZ (IC50 lower than 1 nM) establish simultaneous polar interactions with the hinge and positive regions [27,37,38]. And the polyhalogenated benzimidazole derivative DMAT (IC_50_ = 0.15 μM) establishes two halogen bonds with Glu114 and Val116 of hinge region [39]. While natural derivatives emodin and DBC which only make electrostatic interactions with Lys68 and Glu81 of positive area display moderate inhibitory activity against CK2 (IC_50_ = 2 μM and IC_50_ = 0.1 μM, respectively) [21,40]. It can be found that amino, hydroxyl group and heterocyclic nitrogen atoms tend to establish H-bonds with Glu114 or Val116. The halogen atoms build halogen bond with carbonyl oxygen atom of residue Glu114 and Val116. In contrast, only a few groups such as hydroxyl and carboxylate make electrostatic interactions with Lys68 of the positive area. This suggests that the hinge region plays a pivotal role in the recognition and orientation of CK2 inhibitors [41,42,43,44,45]. As proof of concept, by improving the interaction with the hinge region, trifluoromethylcoumarin derivatives were rationally designed and gave better inhibitory activity compared to the methyl substituted analogues [46]. This will provide the theoretical basis and experiment guidance for the development of anti-cancer drugs by targeting CK2.

### 2.3. Energy Examination

The different binding modes of compounds provide a full and detailed description of each system, while binding energy calculations give qualitative analysis for the effect of inappropriate pharmacophore groups on compound binding affinity. As listed in Table 1, the total ΔG_binding_ of compound **12** (IC_50_ = 0.5 nM) is −46.36 kcal/mol, which is 1.51 kcal/mol and 4.60 kcal/mol lower than that of the compound **27e** and **27h** (IC_50_ > 500 nM, IC_50_ > 1000 nM), respectively. Comparison of the energy component of the two systems shows the favorable formation of complexes is driven by ΔE_ele_, ΔE_vdw_ and ΔG_nonpolar_. The noteworthy energy differences among three systems were that the ΔG_ele_ values of compounds **27e** (−1.50 kcal/mol) and **27h** (−0.28 kcal/mol) exhibited more unfavorable contributions than that of compound **12** (−10.97 kcal/mol), which was in accordance with the crashed H-bond between the deviated compounds with higher RMSD values and the hinge region. However, the non-polar contributions (ΔG_nonpolar_) of three systems shared similar energy components. This may be due to the fact that the wrecked hydrophobic interactions of deviated tricyclic skeleton with residues Val66 and His115 were effectively supplemented by a strong interaction network involving residues Val53 and Ile174.

**Figure 5:**
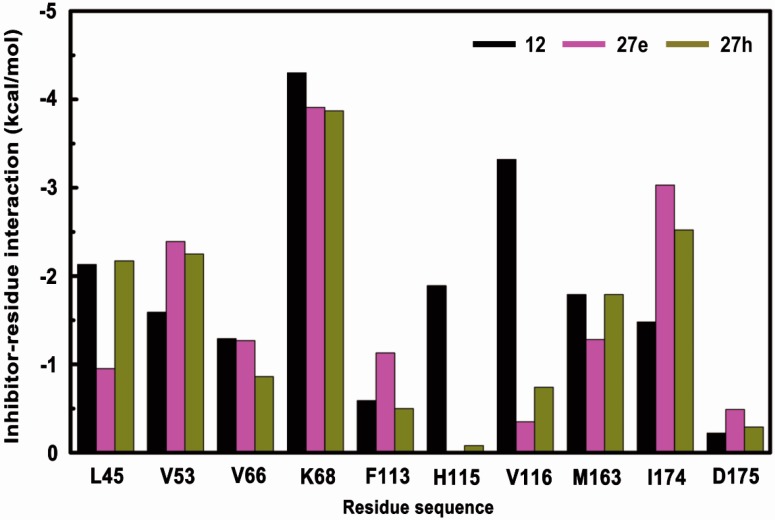
Residue-Based energy decomposition on critical residues for CK2 in complex with compounds **12** (black), **27e** (purple) and **27h** (brown).

Energy decomposition analysis was carried out to evaluate the energetic influences on the contributions of critical residues (Figure 5). Major favorable energy contributions observed in three systems, ranging from −6 to −1 kcal/mol originate predominantly from the hinge region (His115 and Val116), positive region (Lys68 and Asp175) and hydrophobic region（Leu45, Val53, Val66, Phe113, Met163 and Ile174）, which are also reported as the key residues in other CK2-inhibitor complexes. Except His115 and Val116, the energy values of the above mentioned residues, increase to different extents compared with those of the CK2α-**12** system, which indicate that non-2,6-naphtyridine groups exert little influence on the total energy, whereas the contribution of His115 and Val116 in hinge region contribute significantly. These data agree with the abolishment of the conserved H-bond with Val116 and the reorientation of the tricyclic skeleton observed in MD simulation.

## 3. Materials and Methods

### 3.1. Ligands Preparation and Molecular Docking

**Figure 6:**
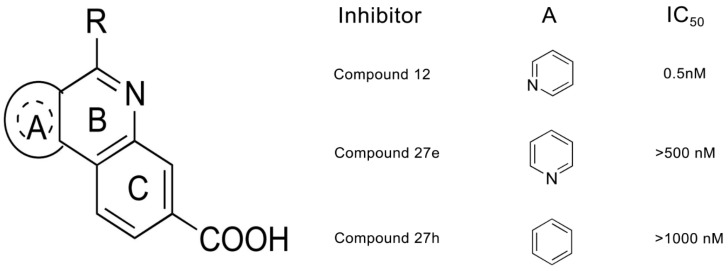
Chemical structures and IC_50_ values of the tricyclic quinolone analogs **12** (R = 3-chlorophenyl), **27e** (R = phenylamino) and **27h** (R = phenylamino).

The four CX-4945 analogues with diverse construction and inhibitory activities IC50 were obtained from the literature [27]. The atomic coordinates of CK2α were retrieved from the Protein Data Bank (PDB ID: 3PE1) [24] and hetero atoms, except for water molecules within 6.5 Å of the compound, were removed. The co-crystallized compound CX-4945 was chosen as a template to build compounds **12**, **27e** and **27h** using Sybyl 8.1 [47,48]. The chemical structures along with biological activities are shown in Figure 6.

The three compounds were docked into the active site of CK2α using the molecular docking program Genetic Optimization for Ligand Docking (GOLD) version 3.0 [49]. In the first step, the compound CX-4945 as the reference structure was applied to locate potential binding sites with amino acids within a 6.5-Å radius of the compound. The crystal structure of CK2α in complex with compound CX-4945 was then used to optimize docking parameters and evaluate the reliability of the docking methods. The standard default genetic algorithm (GA) parameters along with the annealing settings, which optimize the GoldScore fitness function, were employed to predict receptor-ligand binding positions. For each ligand, the early termination criterion was that the top ten ranked solutions were all within 1.5-Å RMSD of each other. The top ranked conformations of all inhibitors clustering in the binding pocket of the protein were directly used to build models. We then employed the binding site in the molecular docking study and the compounds docked were performed to model the other four complexes based on the CK2α-CX-4945 system.

### 3.2. Molecular Dynamics Simulations

Molecular dynamics simulations were carried out using the Amber 10 package [50]. The docked structures of CK2a with compounds **12**, **27e** and **27h** were used as the initial structures for MD calculations. Owing to the lack of parameters needed for the ligands in the Cornell et al. force field [51], the partial atomic charges of the compounds were obtained via quantum electronic structure calculations including an optimization procedure using the Gaussian 03 program [52] at the HF/6-31G* level, electrostatic potential (ESP) generation using the Merz–Singh–Kollman van der Waals parameters [53], and the atom-centered charge fitting through the RESP program implemented in the AMBER 10 package [54]. Subsequently, each system was neutralized by adding Na^+^ ions and then solvated in a truncated octahedral box of TIP3P water molecules with a margin distance of 10 Å [55]. Prior to the simulation, energy minimizations were performed to relieve geometric strain and close intermolecular contacts using the steepest descent and conjugate gradient methods. Each resulting system was gradually heated from 0 to 300 K with weak constraint to the complex (5.0 kcal/mol) over 15 (ps) followed by a constant temperature equilibration at 300 K for 35 ps. Finally, constant pressure dynamics simulations were carried out on the warmed systems for 10 ns in the NPT ensemble, using a non-bonded cutoff of 10 Å to truncate the VDW non-bonded interactions. Temperature (300 K) and constant pressure (1 atm) were maintained by Langevin dynamics temperature coupling with a time constant of 1.0 ps and isotropic position scaling with a relaxation time of 2.0 ps, respectively [56]. The SHAKE algorithm with a tolerance of 10-5 was used to constrain all bonds involving hydrogen atoms [57], allowing a 2 fs time integration step, while the particle-mesh-Ewald (PME) method was introduced for the long-range electrostatic contribution to the force field [58].

### 3.3. MM/PBSA and MM/GBSA Calculations

The binding energies of the three systems were calculated using MM-PB/GBSA methods [59,60]. The binding free energy (ΔG_binding_) was calculated according to the following equation:

ΔG_binding_ = G_complex_ − [G_protein_ + G_ligand_] = ΔE_gas_ + ΔG_sol_ − TΔS (1)

where ΔE_gas_ is the molecular mechanic energy including internal (i.e., bond, angle, and dihedral energies), van der Waals (E_vdw_), and electrostatic energies (E_ele_) was calculated using the sander without applying a cutoff for non-bonded interactions. The free energy of solvation G_sol_ was estimated by continuum solvent methods as the sum of electrostatic (ΔG_polar_) and non-polar (ΔG_nonpolar_) contributions. The electrostatic contribution to the solvation free energy (ΔG_polar_) was calculated by the PB model as implemented in SANDER, applying dielectric constants of 1 and 80 to represent the solute and the exterior medium phases, respectively. The non-polar component (ΔG_nonpolar_), as a linear function of solvent-accessible surface area (SASA) [61], was represented as ΔG_nonpolar_ = λSASA + b, where λ = 0.00542 kcal/(mol Å^2^) and b= 0.92 kcal/mol. Given the large computational overhead and low prediction accuracy, the time consuming conformational entropy change (−TΔS) was not considered [62,63]. The entropy term has been neglected, assuming that it will be very similar for all of the systems. Apart from the calculation of relative binding affinities, a per-residue decomposition of the total energy was performed to evaluate the contribution of each residue to the binding for the three systems, which provided a full description of energetic influences on binding affinity because of carboxylate substituent change. The detailed theory of energy decomposition is described elsewhere.

## 4. Conclusions

Molecular docking, molecular dynamics simulations and energy analysis were employed to explore the pivotal roles of the hinge region sub-pocket in the binding of tricyclic quinolone analogues with CK2. The loss of H-bonds between non-2,6-naphtyridine substituent compounds and the hinge region induce the reorientation of the compound. Meanwhile, the allosteric pathway between the deviated ligands and the affected positions is proposed. In addition, the energy analysis enables the qualitative investigation of the effects of non-2,6-naphtyridinesubstituent changes on ligand binding affinity. The most important presented here is a summary of known polar pharmacophore groups of ATP-competitive inhibitors. The findings of this study will be valuable for future rational design of novel CK2 inhibitors as promising anticancer therapeutics.

## Figures and Tables

**Table 1:** Energy terms of MM/GBSA results for three CK2α-inhibitor complexes systems.

Energy Term (kcal/mol)	12	27e	27h
ΔE_ele_	−100.93 ± 4.0	−112.33 ± 3.3	−93.01 ± 4.0
ΔE_vdw_	−30.55 ± 3.0	−37.76 ± 3.1	−36.28 ± 2.5
ΔE_gas_^a^	−131.48 ± 6.0	−150.10 ± 5.7	−129.29 ± 3.4
ΔG_nonpolar_	−4.84 ± 0.12	−5.59 ± 0.20	−5.20 ± 0.10
ΔG_polar_	89.96 ± 3.9	110.84 ± 3.6	92.73 ± 5.0
ΔG_sol_^b^	85.12 ± 3.9	105.25 ± 2.2	87.52 ± 3.5
ΔG_ele_^c^	−10.97 ± 2.7	−1.50 ± 3.1	−0.28 ± 2.2
ΔG_binding_^d^	−46.36 ± 3.2	−44.85 ± 3.1	−41.76 ± 3.0
ΔΔG_binding_	0	1.51	4.60

^a^ ΔE_gas_ = ΔE_ele_ + ΔE_vdw_; ^b^ ΔG_sol_ = ΔG_polar_ + ΔG_nonpolar_; ^c^ ΔG_ele_ = ΔE_ele_ + ΔG_polar_; ^d^ ΔG_binding_ = ΔE_ele_ + ΔE_vdw_ + ΔG_sol_.

## References

[1] Ruzzene M., Pinna L.A. (2010). Addiction to protein kinase CK2: A common denominator of diverse cancer cells?. Biochim. Biophys. Acta.

[2] Trembley J.H., Wang G., Unger G., Slaton J., Ahmed K. (2009). Protein kinase CK2 in health and disease: CK2: A key player in cancer biology. Cell. Mol. Life Sci..

[3] Meggio F., Pinna L.A. (2003). One-Thousand-and-One substrates of protein kinase CK2?. FASEB J. Off. Publ. Fed. Am. Soc. Exp. Biol..

[4] Filhol O., Martiel J.L., Cochet C. (2004). Protein kinase CK2: A new view of an old molecular complex. EMBO Rep..

[5] Cozza G., Pinna L.A. (2016). Casein kinases as potential therapeutic targets. Expert Opin. Ther. Targets.

[6] Guerra B., Issinger O.G. (2008). Protein kinase CK2 in human diseases. Curr. Med. Chem..

[7] Ortega C.E., Seidner Y., Dominguez I. (2014). Mining CK2 in cancer. PLoS ONE.

[8] Filhol O., Giacosa S., Wallez Y., Cochet C. (2015). Protein kinase CK2 in breast cancer: The CK2β regulatory subunit takes center stage in epithelial plasticity. Cell. Mol. Life Sci..

[9] Swider R., Maslyk M., Martin-Santamaria S., Ramos A., de Pascual-Teresa B. (2011). Multisite-Directed inhibitors of protein kinase CK2: New challenges. Mol. Cell. Biochem..

[10] Swider R., Maslyk M., Zapico J.M., Coderch C., Panchuk R., Skorokhyd N., Schnitzler A., Niefind K., de Pascual-Teresa B., Ramos A. (2015). Synthesis, biological activity and structural study of new benzotriazole-based protein kinase CK2 inhibitors. Rsc Adv..

[11] Cozza G., Bortolato A., Moro S. (2010). How druggable is protein kinase CK2?. Med. Res. Rev..

[12] Battistutta R. (2009). Protein kinase CK2 in health and disease: Structural bases of protein kinase CK2 inhibition. Cell. Mol. Life Sci..

[13] Perez D.I., Gil C., Martinez A. (2011). Protein kinases CK1 and CK2 as new targets for neurodegenerative diseases. Med. Res. Rev..

[14] Pagano M.A., Bain J., Kazimierczuk Z., Sarno S., Ruzzene M., Di Maira G., Elliott M., Orzeszko A., Cozza G., Meggio F. (2008). The selectivity of inhibitors of protein kinase CK2: An update. Biochem. J..

[15] Sarno S., Papinutto E., Franchin C., Bain J., Elliott M., Meggio F., Kazimierczuk Z., Orzeszko A., Zanotti G., Battistutta R. (2011). ATP site-directed inhibitors of protein kinase CK2: An update. Curr. Top. Med. Chem..

[16] Li Y., Luan Y., Qi X., Li M., Gong L., Xue X., Wu X., Wu Y., Chen M., Xing G. (2010). Emodin triggers DNA double-strand breaks by stabilizing topoisomerase II-DNA cleavage complexes and by inhibiting ATP hydrolysis of topoisomerase II. Toxicol. Sci..

[17] Siddiqui-Jain A., Drygin D., Streiner N., Chua P., Pierre F., O’Brien S.E., Bliesath J., Omori M., Huser N., Ho C. (2010). CX-4945, an orally bioavailable selective inhibitor of protein kinase CK2, inhibits prosurvival and angiogenic signaling and exhibits antitumor efficacy. Cancer Res..

[18] Senhwa Biosciences, Inc. Senhwa Biosciences CX-4945 Granted Orphan Drug Designation by the US FDA in Cholangiocarcinoma. http://www.prnewswire.com/news-releases/senhwa-biosciences-cx-4945-granted-orphan-drug-designation-by-the-us-fda-in-cholangiocarcinoma-300385278.html.

[19] Zanin S., Borgo C., Girardi C., O’Brien S.E., Miyata Y., Pinna L.A., Donella-Deana A., Ruzzene M. (2012). Effects of the CK2 inhibitors CX-4945 and CX-5011 on drug-resistant cells. PLoS ONE.

[20] Siddiqui-Jain A., Bliesath J., Macalino D., Omori M., Huser N., Streiner N., Ho C.B., Anderes K., Proffitt C., O’Brien S.E. (2012). CK2 inhibitor CX-4945 suppresses DNA repair response triggered by DNA-targeted anticancer drugs and augments efficacy: Mechanistic rationale for drug combination therapy. Mol. Cancer Ther..

[21] Chilin A., Battistutta R., Bortolato A., Cozza G., Zanatta S., Poletto G., Mazzorana M., Zagotto G., Uriarte E., Guiotto A. (2008). Coumarin as attractive casein kinase 2 (CK2) inhibitor scaffold: An integrate approach to elucidate the putative binding motif and explain structure-activity relationships. J. Med. Chem..

[22] Battistutta R., Mazzorana M., Sarno S., Kazimierczuk Z., Zanotti G., Pinna L.A. (2005). Inspecting the structure-activity relationship of protein kinase CK2 inhibitors derived from tetrabromo-benzimidazole. Chem. Boil..

[23] Haddach M., Pierre F., Regan C.F., Borsan C., Michaux J., Stefan E., Kerdoncuff P., Schwaebe M.K., Chua P.C., Siddiqui-Jain A. (2012). Synthesis and SAR of inhibitors of protein kinase CK2: Novel tricyclic quinoline analogs. Bioorg. Med. Chem. Lett..

[24] Battistutta R., Cozza G., Pierre F., Papinutto E., Lolli G., Sarno S., O’Brien S.E., Siddiqui-Jain A., Haddach M., Anderes K. (2011). Unprecedented selectivity and structural determinants of a new class of protein kinase CK2 inhibitors in clinical trials for the treatment of cancer. Biochemistry.

[25] Zhou Y., Zhang N., Zhong R.G. (2013). Exploring the crucial structural elements required for tricyclic quinoline analogs as protein kinase CK2 inhibitors by a combined computational analysis. Med. Chem. Res..

[26] Zhou Y., Li X., Zhang N., Zhong R. (2015). Structural basis for low-affinity binding of non-R2 carboxylate-substituted tricyclic quinoline analogs to CK2alpha: Comparative molecular dynamics simulation studies. Chem. Biol. Drug Des..

[27] Pierre F., Chua P.C., O’Brien S.E., Siddiqui-Jain A., Bourbon P., Haddach M., Michaux J., Nagasawa J., Schwaebe M.K., Stefan E. (2011). Discovery and SAR of 5-(3-chlorophenylamino)benzo[*c*][2,6]naphthyridine-8-carboxylic acid (CX-4945), the first clinical stage inhibitor of protein kinase CK2 for the treatment of cancer. J. Med. Chem..

[28] Borhani D.W., Shaw D.E. (2012). The future of molecular dynamics simulations in drug discovery. J. Comput.-Aided Mol. Des..

[29] Durrant J.D., McCammon J.A. (2011). Molecular dynamics simulations and drug discovery. BMC Biol..

[30] Li J., Du Y., Liu X., Shen Q.C., Huang A.L., Zheng M.Y., Luo X.M., Jiang H.L. (2013). Binding sensitivity of adefovir to the polymerase from different genotypes of HBV: Molecular modeling, docking and dynamics simulation studies. Acta Pharmacol. Sin..

[31] Zhang N., Zhong R. (2010). Structural basis for decreased affinity of Emodin binding to Val66-mutated human CK2α as determined by molecular dynamics. J. Mol. Model..

[32] Karplus M., McCammon J.A. (2002). Molecular dynamics simulations of biomolecules. Nat. Struct. Biol..

[33] Jin H.X., Wu T.X., Jiang Y.J., Zou J.W., Zhuang S.L., Mao X., Yu Q.S. (2007). Role of phosphorylated Thr-197 in the catalytic subunit of cAMP-dependent protein kinase. J. Mol. Struct. THEOCHEM.

[34] Welburn J.P., Tucker J.A., Johnson T., Lindert L., Morgan M., Willis A., Noble M.E., Endicott J.A. (2007). How tyrosine 15 phosphorylation inhibits the activity of cyclin-dependent kinase 2-cyclin A. J. Biol. Chem..

[35] Zhang N., Jiang Y., Zou J., Yu Q., Zhao W. (2009). Structural basis for the complete loss of GSK3β catalytic activity due to R96 mutation investigated by molecular dynamics study. Proteins.

[36] Papinutto E., Ranchio A., Lolli G., Pinna L.A., Battistutta R. (2012). Structural and functional analysis of the flexible regions of the catalytic α-subunit of protein kinase CK2. J. Struct. Biol..

[37] Nie Z., Perretta C., Erickson P., Margosiak S., Almassy R., Lu J., Averill A., Yager K.M., Chu S. (2007). Structure-Based design, synthesis, and study of pyrazolo[1,5-*a*][1,3,5]triazine derivatives as potent inhibitors of protein kinase CK2. Bioorg. Med. Chem. Lett..

[38] Dowling J.E., Chuaqui C., Pontz T.W., Lyne P.D., Larsen N.A., Block M.H., Chen H., Su N., Wu A., Russell D. (2012). Potent and selective inhibitors of CK2 kinase identified through structure-guided hybridization. ACS Med. Chem. Lett..

[39] Pagano M.A., Meggio F., Ruzzene M., Andrzejewska M., Kazimierczuk Z., Pinna L.A. (2004). 2-Dimethylamino-4,5,6,7-tetrabromo-1*H*-benzimidazole: A novel powerful and selective inhibitor of protein kinase CK2. Biochem. Biophys. Res. Commun..

[40] Yim H., Lee Y.H., Lee C.H., Lee S.K. (1999). Emodin, an anthraquinone derivative isolated from the rhizomes of *Rheum palmatum*, selectively inhibits the activity of casein kinase II as a competitive inhibitor. Planta Med..

[41] Kinoshita T., Sekiguchi Y., Fukada H., Nakaniwa T., Tada T., Nakamura S., Kitaura K., Ohno H., Suzuki Y., Hirasawa A. (2011). A detailed thermodynamic profile of cyclopentyl and isopropyl derivatives binding to CK2 kinase. Mol. Cell. Biochem..

[42] Nakanishi I., Murata K., Nagata N., Kurono M., Kinoshita T., Yasue M., Miyazaki T., Takei Y., Nakamura S., Sakurai A. (2015). Identification of protein kinase CK2 inhibitors using solvent dipole ordering virtual screening. Eur. J. Med. Chem..

[43] Ohno H., Minamiguchi D., Nakamura S., Shu K., Okazaki S., Honda M., Misu R., Moriwaki H., Nakanishi S., Oishi S. (2016). Structure-Activity relationship study of 4-(thiazol-5-yl)benzoic acid derivatives as potent protein kinase CK2 inhibitors. Bioorg. Med. Chem..

[44] Dowling J.E., Alimzhanov M., Bao L., Chuaqui C., Denz C.R., Jenkins E., Larsen N.A., Lyne P.D., Pontz T., Ye Q. (2016). Potent and selective CK2 kinase inhibitors with effects on Wnt pathway signaling in vivo. ACS Med. Chem. Lett..

[45] Ostrynska O.V., Balanda A.O., Bdzhola V.G., Golub A.G., Kotey I.M., Kukharenko O.P., Gryshchenko A.A., Briukhovetska N.V., Yarmoluk S.M. (2016). Design and synthesis of novel protein kinase CK2 inhibitors on the base of 4-aminothieno[2,3-*d*]pyrimidines. Eur. J. Med. Chem..

[46] Zhang N., Chen W.J., Zhou Y., Zhao H., Zhong R.G. (2016). Rational design of coumarin derivatives as CK2 inhibitors by improving the interaction with the hinge region. Mol. Inform..

[47] Clark M., Cramer R.D., Vanopdenbosch N. (1989). Validation of the general-purpose Tripos 5.2 force-field. J. Comput. Chem..

[48] SYBYL, V. (2008). SYBYL, Version 8.1.

[49] Jones G., Willett P., Glen R.C., Leach A.R., Taylor R. (1997). Development and validation of a genetic algorithm for flexible docking. J. Mol. Biol..

[50] Case D.A., Cheatham T.E., Darden T., Gohlke H., Luo R., Merz K.M., Onufriev A., Simmerling C., Wang B., Woods R.J. (2005). The Amber biomolecular simulation programs. J. Comput. Chem..

[51] Cornell W.D., Cieplak P., Bayly C.I., Gould I.R., Merz K.M., Ferguson D.M., Spellmeyer D.C., Fox T., Caldwell J.W., Kollman P.A. (1995). A second generation force-field for the simulation of proteins, nucleic-acids, and organic-molecules. J. Am. Chem. Soc..

[52] Gaussian 03, R.C. (2003). Gaussian 03, Revision C.02.

[53] Besler B.H., Merz K.M., Kollman P.A. (1990). Atomic charges derived from semiempirical methods. J. Comput. Chem..

[54] Fox T., Kollman P.A. (1998). Application of the RESP methodology in the parametrization of organic solvents. J. Phys. Chem. B.

[55] Jorgensen W.L., Chandrasekhar J., Madura J.D., Impey R.W., Klein M.L. (1983). Comparison of simple potential functions for simulating liquid water. J. Chem. Phys..

[56] Berendsen H.J.C., Postma J.P.M., Vangunsteren W.F., Dinola A., Haak J.R. (1984). Molecular-Dynamics with coupling to an external bath. J. Chem. Phys..

[57] Ryckaert J.-P., Ciccotti G., Berendsen H.J.C. (1977). Numerical integration of the cartesian equations of motion of a system with constraints: Molecular dynamics of *n*-alkanes. J. Comput. Phys..

[58] Darden T., York D., Pedersen L. (1993). Particle mesh Ewald: An *N*⋅log(*N*) method for Ewald sums in large systems. J. Chem. Phys..

[59] Massova I., Kollman P.A. (2000). Combined molecular mechanical and continuum solvent approach (MM-PBSA/GBSA) to predict ligand binding. Perspect. Drug Discov. Des..

[60] Kollman P.A., Massova I., Reyes C., Kuhn B., Huo S., Chong L., Lee M., Lee T., Duan Y., Wang W. (2000). Calculating structures and free energies of complex molecules: Combining molecular mechanics and continuum models. Acc. Chem. Res..

[61] Sitkoff D., Sharp K.A., Honig B. (1994). Accurate calculation of hydration free energies using macroscopic solvent models. J. Phys. Chem..

[62] Wang W., Kollman P.A. (2001). Computational study of protein specificity: The molecular basis of HIV-1 protease drug resistance. Proc. Natl. Acad. Sci. USA.

[63] Wang J., Morin P., Wang W., Kollman P.A. (2001). Use of MM-PBSA in reproducing the binding free energies to HIV-1 RT of TIBO derivatives and predicting the binding mode to HIV-1 RT of efavirenz by docking and MM-PBSA. J. Am. Chem. Soc..

